# Prevalence and Characteristics of Chronic Pain in Buprenorphine and Methadone-Maintained Patients

**DOI:** 10.3389/fpsyt.2021.641430

**Published:** 2021-04-26

**Authors:** Jessica Delorme, Lucie Pennel, Georges Brousse, Jean-Pierre Daulouède, Jean-Michel Delile, Philippe Lack, Antoine Gérard, Maurice Dematteis, Jean-Luc Kabore, Nicolas Authier, Chouki Chenaf, TOXIDOL Study group

**Affiliations:** ^1^Université Clermont Auvergne, CHU Clermont-Ferrand, Inserm, Neuro-Dol, Service de Pharmacologie médicale, Centres Addictovigilance et Pharmacovigilance, Centre Evaluation et Traitement de la Douleur, Service Psychiatrie-Addictologie, Clermont-Ferrand, France; ^2^Observatoire Français des Médicaments Antalgiques (OFMA) / French monitoring centre for analgesic drugs, Université Clermont Auvergne - CHU Clermont-Ferrand, Clermont-Ferrand, France; ^3^Service d'Addictologie, CHU Grenoble Alpes, Université Grenoble Alpes, Grenoble, France; ^4^Centre de Soins et d'Accompagnement et de Prévention en Addictologie (CSAPA), BIZIA, Médecins du Monde, Centre Hospitalier de la côte Basque, Bayonne, France; ^5^Centre de Soins et d'Accompagnement et de Prévention en Addictologie (CSAPA) “Maurice Serisé”, Comité d'Etude et d'Information sur la Drogue (CEID), Bordeaux, France; ^6^Centre de Soins et d'Accompagnement et de Prévention en Addictologie (CSAPA), Centre Hospitalier de la Croix Rousse, Lyon, France; ^7^Centre de Soins et d'Accompagnement et de Prévention en Addictologie (CSAPA), Centre Hospitalier Emile Roux, Le Puy-en-Velay, France; ^8^Institut Analgesia, Faculté de Médecine, BP38, Clermont-Ferrand, France

**Keywords:** methadone, buprenorphine, chronic pain, prevalence, epidemiology, pain management, opioid agonist treatment

## Abstract

Chronic pain and substance use disorders frequently co-occur. Indeed, chronic pain is highly prevalent, affecting 23–68% of patients receiving opioid agonist treatments (OAT) worldwide. The majority of available estimates come from American studies, but data are still lacking in Europe. We aim to provide European estimates of the prevalence of chronic pain in patients receiving OAT using French data, since France is the first European country in terms of number of patients with OAT. The secondary objectives were to characterize the features and management of chronic pain, as well identify associated risk factors. We conducted a multicenter, cross-sectional study, recruiting patients treated either with buprenorphine or methadone in 19 French addiction centers, from May to July 2016. All participants had to complete a semi-directed questionnaire that collected sociodemographic and medical data, pain characteristics, and licit or illicit drug consumption. In total, 509 patients were included. The prevalence of chronic pain was estimated at 33.2% (95% CI: 29.1–37.3). Compared to non-chronic pain patients, chronic pain patients were older (38.4 vs. 36.1 years, *p* = 0.006), were more unemployed (66 vs. 52%, *p* = 0.003), had more psychiatric comorbidities (50 vs. 39%, *p* = 0.02), and split their OAT for pain management more frequently (24 vs. 7%, *p* = 0.009). Pain intensity was moderate or severe in 75% of chronic pain patients. Among patients with chronic pain, 15.4% were not prescribed, and did not self-medicate with, any analgesic drugs, 52.1% were prescribed analgesics (non-opioid analgesics, 76.3%; codeine, tramadol, opium, 27.2%; and morphine, fentanyl, oxycodone, 11.8%), and 32.5% exclusively self-medicated with analgesics. Moreover, 20.1% of patients with chronic pain also used illicit drugs for pain relief. On multivariate analysis, variables that remained significantly associated with chronic pain were age [OR = 1.03 (95% CI: 1.00–1.05], *p* = 0.02], anxiety [OR = 1.52 (1.15–2.02), *p* = 0.003], and depression [OR = 1.25 (1.00–1.55), *p* = 0.05]. Chronic pain is a highly prevalent condition in patients receiving OAT, and its appropriate management remains uncertain, since insufficient relief and frequent additional self-medications with analgesics or illicit drugs were reported by these patients. Increased awareness among caregivers is urgently needed regarding a systematic and careful assessment, along with an adequate management of chronic pain in patients receiving OAT.

## Introduction

Chronic pain and opioid use disorders are frequently intertwined and represent a worldwide health problem. Indeed, the prevalence of chronic pain is higher in patients receiving opioid agonist treatment (OAT), ranging from 23 to 68% (1–10), while, in the general population, the chronic pain prevalence range from 8.7 to 64.4%, with a pooled mean of 31% (IC 95%: 30.8–31.2) worldwide according to a recent meta-analysis (11). Moreover, pain intensity is more frequently moderate to severe in patients receiving OAT (23.4–44%) (1, 2, 5, 7) compared to the general population (12–29%) (11). Chronic pain has also been associated with severe medical and psychiatric comorbidities in patients receiving OAT (1–3, 7, 12–14). Of these comorbidities, pain-related sleep disorders were the greatest problem in patients receiving OAT (73% of methadone patients with chronic pain), followed by affective, physical activity, and social relationship dysfunction (1).

Treating both pain and addiction proves to be a significant challenge, and caregivers commonly report that all their diagnostic and therapeutic decisions are subject to ambiguity (15). Some studies also showed that clinicians in opioid use disorder treatment settings often felt unprepared to treat chronic pain due to a lack of expertise and of referrals in managing co-occurring chronic pain and opioid use disorder (16, 17). Moreover, many studies showed that patients receiving OAT perceived that their pain was inadequately managed (18–21). Chronic pain in patients receiving OAT is even more problematic since inappropriate pain management may lead to a subsequent use of licit or illicit opioids (22), such as prescribed opioids in addition to heroin for self-management of pain (1, 7), and contribute to return to non-medical opioid use. This is of particular concern in the context of the still ongoing US opioid epidemic (23). Therefore, it is essential to precisely estimate the prevalence of chronic pain in patients receiving OAT.

The vast majority of available data are from American studies. As far as we know, no European data are currently available. In 2018, while 680,040 opioid users received an opioid agonist treatment in Europe, more than 178,000 were French patients treated either by buprenorphine±naloxone (65%), or methadone (35%) (24, 25). We propose to provide European estimates of chronic pain prevalence in patients receiving OAT using French data, since France represents the first European country in terms of number of patients and accounts for more than one quarter of patients receiving OAT in Europe.

Thus, the aim of this study was to estimate the prevalence of chronic pain in patients receiving OAT, to characterize chronic pain, to describe analgesics treatments, and to identify associated risk factors.

## Methods

### Study Design and Setting

This was a cross-sectional study conducted on patients receiving OAT treated either with methadone or buprenorphine. Patients were recruited from 19 addiction centers in France, from May to July 2016. In France, all registered medical doctors are allowed to prescribe buprenorphine without any special education or licensing; regulations allow buprenorphine prescriptions of up to 28 days of take-home doses. In contrast, treatment with methadone is less accessible and requires mandatory initiation within a specialized addiction center or health care facility, and management of the patient can only be transferred to a non-specialist physician once the patient has been stabilized; up to 14 days (syrup) or 28 days (capsule) supply at a time may be prescribed.

During the recruitment period, all patients seen in consultation were systematically proposed to participate in the study.

### Participants

To fit the inclusion criteria, all patients were over 18 years, and being treated with methadone or buprenorphine for opioid use disorders. Exclusion criteria were an inability to understand the patients' information and informed consent form, and an undergoing measure of legal protection. All participants received oral and written information which described the study. All participants gave oral consent and their participation was totally voluntary and anonymous.

### Measures

The questionnaire was semi-directed and given during a routine medical consultation. The questionnaire consisted of five parts collecting the following information:

1. Demographic data on gender, age, familial situation, and professional situation.
2. Medical data, on human immunodeficiency virus (HIV), hepatitis B virus (HBV), and hepatitis C virus (HCV) infection status, consumption of tobacco or alcohol, type (methadone or buprenorphine) and duration of OAT, form of methadone treatment (syrup or capsule), daily dose of OAT, OAT dose splitting, age of the first illicit opioid use, and concomitant psychotropic drug use (antidepressants, antipsychotics, mood stabilizers, anxiolytics, hypnotics) were collected according to the medical record and the patient's statement.
3. Chronic pain was defined as any pain experienced for at least 3 months using the following question: “how long have you been experiencing this pain? (<3 months; 3–6 months; 7–12 months; 1–5 years; or >5 years), excluding patients with answer <3 months.” Then, the Brief Pain Inventory (BPI) was administered to assess the characteristics of chronic pain (intensity and location) and interferences in daily life (26). The BPI also evaluates pain severity at its “worst,” “least,” “average,” and “now” (current pain), measured with a numeric scale (NS) (no pain = 0, unbearable pain = 10). According to the average pain score, pain intensity was classified as mild pain (NS = 1–4), moderate pain (NS = 5–6) and severe pain (NS = 7–10). Pain interference was assessed in seven domains of work, walking, sleep, relationship, enjoyment, moods and general activity by NS (no pain interference = 0, complete pain interference = 10). Interference was considered present when a patient reported a score of 5 or higher on the “interference” item. One extra question of “Have you felt pain associated with opioid withdrawal during the last 7 days?” was added to assess withdrawal-related pain (patients were specifically asked about the occurrence of withdrawal-related painful symptoms such as abdominal cramps, muscle twitches, or diffuse aching of bone, joints, and muscles).
4. Information on licit (acetaminophen, non-steroidal anti-inflammatory drugs, nefopam, analgesic opioids, and benzodiazepines) and illicit drugs used to treat pain during the previous 3 months (prescription, over the counter (OTC) and street) was also collected.
5. Psychiatric disorders were measured with a 6-item validated version of the Symptom Checklist-90 (SCL-90) (27). This brief self-report instrument (SCL-6) provides an assessment of past 30-day psychiatric functioning, including anxiety, depression, and psychoticism; each of these three items is rated on a five-point Likert-scale of distress, ranging from“not at all” (0) to “extremely.”

### Statistical Analysis

Data were expressed as frequencies and associated percentages for categorical data and as mean ± standard deviation for quantitative variables. The analysis was conducted using descriptive statistics to compare the characteristics of patients receiving OAT with and without chronic pain. The Chi-square test was used to compare categorical variables, while the Student's *t*-test was used to compare quantitative variables. To examine the factors associated with chronic pain in patients receiving OAT, a univariate logistic regression analysis was first performed, followed by a multivariate logistic regression analysis, after checking that basic statistical assumptions were met (independence of observations, linearity in the logit for the independent variables, absence of multicollinearity among the independent variables, and lack of strongly influential outliers). Factors was considered significant in univariate analysis (entered into the model if *p* < 0.25) and accordingly to clinically relevant variables such as age and gender. Associated *p*-values were computed with corresponding odds ratios (OR), or adjusted OR, and 95% confidence intervals (95% CI). All statistical analyses were performed using SAS for Windows version 9.3 (SAS Institute, North Carolina, USA).

### Ethical Review

This research obtained the approval of the French Research Ethics Committee (CPP Sud-Est 6) on 26th February 2016.

## Results

### General Characteristics of Patients Receiving OAT

During the 3-month recruitment period, the questionnaire was given to 621 patients receiving OAT and a total of 509 agreed to participate in the study (response rate 82%). Of the participants, 166 were buprenorphine-maintained patients (BMPs) and 343 were methadone-maintained patients (MMPs). In patients receiving OAT, the mean age was 36.9 ± 9.2 years, the majority were male (77.8%), single (62.4%), and unemployed (56.5%) (Table 1). The mean dose of buprenorphine was 9.7 ± 6.6 mg/day (min: 0.4 - max: 32) and 44.0% were treated with brand-name buprenorphine (Subutex®). The mean dose of methadone was 63.6 ± 40.5 mg/day (2–300) and the majority of patients had the syrup form (58.6%). More than one fifth of patients receiving OAT (22.1%) split their OAT, mainly to manage withdrawal symptoms (59.1%). Concomitant psychotropic drugs were used by 42.8% of patients receiving OAT. In terms of infection status, 3.2% of patients receiving OAT had HIV, 19.2% had HCV and 5% had HBV. The majority of patients were active smokers, and 24.0% drank more than three glasses of alcohol per day.

**Table 1:** General characteristics of patients receiving OAT.

	**Patients receiving OAT *n* = 509**	**Patients with buprenorphine *n* = 166**	**Patients with methadone *n* = 343**	*P*-value^*^
	**%**	**%**	**%**	
**Age mean ± SD [min-max]**	36.9 ± 9.2 [19–64]	38.3 ± 9.2 [19–64]	36.2 ± 9.1 [19–62]	0.01
**Male gender**	77.8	74.7	79.3	0.24
**Couple**	37.6	38.6	37.1	0.74
**Unemployment**	56.5	49.4	60.0	0.02
**Type of OAT**				
Buprenorphine	32.6	—	—	
Methadone	67.4	—	—	
**Duration of OAT**				
<1 year	16.4	15.4	16.8	0.70
1–5 years	47.5	46.0	48.2	
>5 years	36.1	38.6	35.0	
**OAT Dose (mg/day), mean ± SD [min-max]**	–	9.7 ± 6.6 [0.4–32]	63.6 ± 40.5 [2–300]	—
**Type of buprenorphine**				
Subutex®	—	44.0	—	—
Generic	—	35.5	—	—
Suboxone®	—	20.5	—	—
**Type of methadone**				
Syrup	—	—	58.6	—
Capsule	—	—	41.4	—
**OAT Splitting**	22.1	32.3	17.2	<0.0001
**Reasons for OAT splitting**				
Withdrawal	59.1	35.8	44.1	0.38
Anxiety	34.6	38.5	31.0	0.41
Pain	12.7	5.8	18.9	0.03
Sleep	24.6	11.5	36.2	0.003
**Psychotropic drug use**	42.8	50.0	39.4	0.02
**HIV**	3.2	3.1	3.3	0.99
Ongoing treatment	81.3	100	72.7	
**HCV**	19.2	18.3	19.6	0.93
Ongoing treatment	10.6	13.3	9.4	
**HBV**	5.0	4.3	5.4	0.86
Ongoing treatment	4.0	0	5.6	
**Tobacco**	87.8	87.4	88.1	0.82
**Alcohol consumption ≥ 3 glasses/day**	24.0	17.5	27.3	0.02
**Age at first illicit opioid consumption, mean ± SD [min-max]**	20.3 ± 5.1 [11–47]	21.1 ± 5.7 [11–47]	19.9 ± 5.1 [12–47]	0.02
**SCL_6 score, mean ± SD [min-max]**	1.06 ± 0.92 [0–4]	1.02 ± 0.92 [0–4]	1.07 ± 0.93 [0–3.8]	0.55
Anxiety	0.92 ± 0.98 [0–4]	0.92 ± 0.96 [0–4]	0.93 ± 0.99 [0–4]	0.95
Depression	1.32 ± 1.13 [0–4]	1.25 ± 1.10 [0–4]	1.36 ± 1.14 [0–4]	0.32
Psychoticism	0.91 ± 1.03 [0–4]	0.89 ± 1.04 [0–4]	0.93 ± 1.03 [0–4]	0.64

* *Comparisons between patients with buprenorphine and patients with methadone*.

*OAT, opioid agonist treatment; HIV, human immunodeficiency virus; HCV, hepatitis C virus; HBV, hepatitis B virus*.

### Prevalence of Pain in Patients Receiving OAT

The prevalence of chronic pain in patients receiving OAT was 33.2% (29.1–37.3), with no difference seen between MMP and BMP groups (Table 2). The prevalence of chronic pain in patients receiving OAT increased with age, from 28.3% in those aged <25 years to 36.6% in the 50–64 age group.

**Table 2:** Prevalence of chronic pain in patients receiving OAT.

	**Patients receiving OAT *n* = 509**	**Patients with buprenorphine *n*= 166**	**Patients with methadone *n* = 343**	*P*-value^*^
	**%**	**%**	**%**	
**Chronic pain** [95% CI]	33.2 [29.1–37.3]	36.8 [29.5–44.1]	31.5 [26.6–36.4]	0.24
**Chronic pain according to age group**				
*<25*	*28.3*	*27.3*	*28.6*	–
*[25–34]*	*27.8*	*27.3*	*27.9*	
*[35–49]*	*37.4*	*41.1*	*35.1*	
*[50–64]*	*36.6*	*43.8*	*32.0*	
**Withdrawal-related pain during last 8 days**	17.3	14.6	18.8	0.24
**First consumption of illicit opioid in painful context**	8.9	7.3	9.7	0.39

* *Comparisons between patients with buprenorphine and patients with methadone*.

*OAT, opioid agonist treatment; HIV, human immunodeficiency virus; HCV, hepatitis C virus; HBV, hepatitis B virus. Italic values were expressed in years*.

The prevalence of withdrawal-related pain in patients receiving OAT was 17.3% (14.0–20.6). Patients with a withdrawal-related pain consumed significantly more street drugs than patients without withdrawal-related pain [street buprenorphine (5.0 vs. 0.5%, *p* = 0.001), street methadone (3.0 vs. 0.7%, *p* = 0.03), heroin (21.0 vs. 10.0%, *p* = 0.005), street morphine (8.0 vs. 3.0%, *p* = 0.01)]. Compared to patients receiving OAT with no chronic pain, the prevalence of withdrawal-related pain in patients receiving OAT with chronic pain was higher, 21.9% (15.7–28.1) vs. 15.1% (11.3–18.90), *p* = 0.05.

### Characteristics, Intensity, and Daily Life Interference of Chronic Pain

In patients receiving OAT with chronic pain, 18.5% had experienced chronic pain for <1 year, 35.2% for 1–5 years and 46.3% for more than 5 years. Half of patients receiving OAT with chronic pain had back pain and 36.1% had lower limb pain. The average pain intensity was 4.9 ± 2.3 out of 10, where 25.0% of patients receiving OAT with chronic pain had mild pain, 54.0% had moderate pain and 21.0% had severe pain. Among patients receiving OAT with moderate or severe pain, 87.1% had an analgesic treatment.

**Figure 1:**
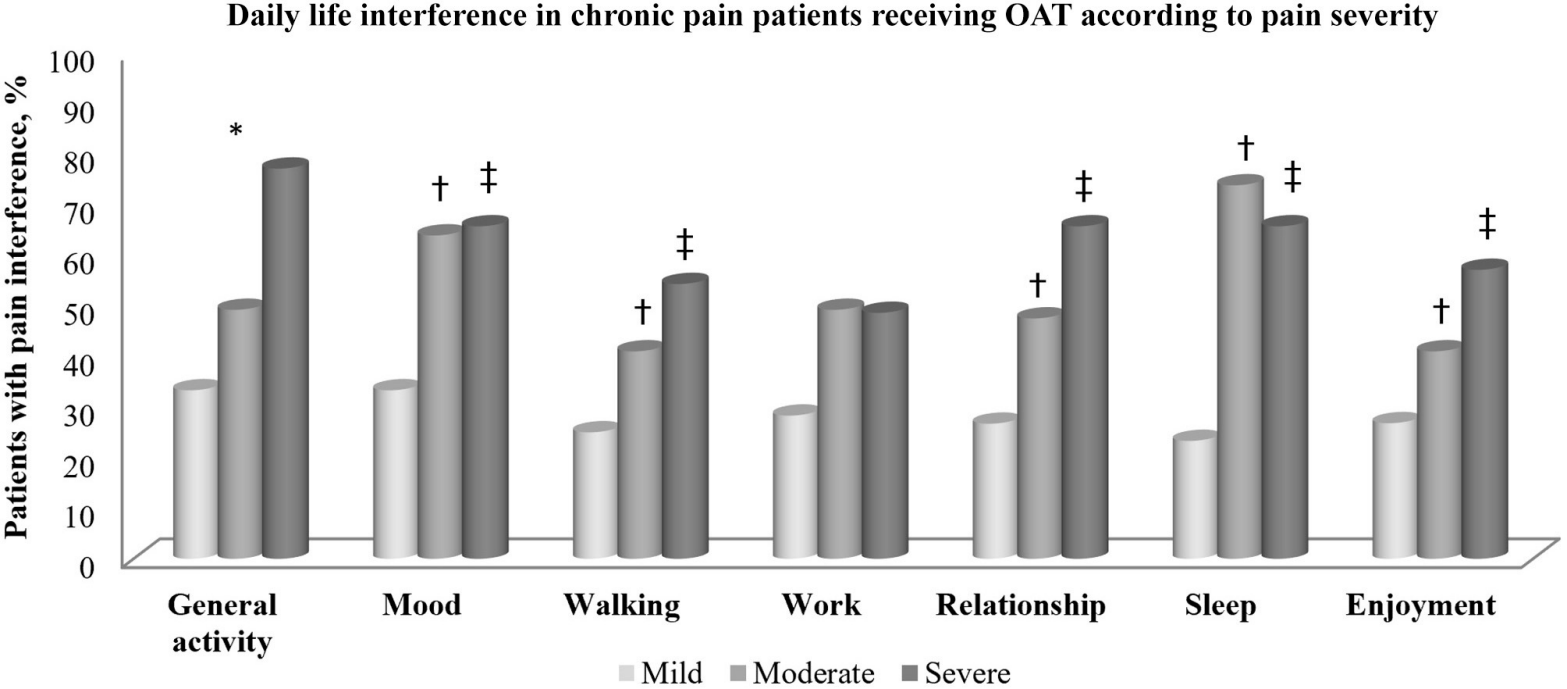
Daily life interference in chronic pain patients according to pain severity. *All groups different, *p* < 0.01. ^†^Difference between moderate pain vs. mild pain; *p* < 0.01. ^‡^Difference between severe pain vs. mild pain; *p* < 0.01.

There was a significant relationship between the degree of pain interference and the intensity of pain, except for the “work” item (Figure 1). Interference with general activity differed significantly in all three subgroups (mild, moderate, and severe pain), whereas the impact on mood, walking, relationship, sleep and enjoyment differed only between mild and moderate or severe pain.

### Characteristics of Patients Receiving OAT With Chronic Pain

Compared to patients receiving OAT with no chronic pain, patients receiving OAT with chronic pain were significantly slightly older, had a higher rate of unemployment, a higher incidence of psychiatric disorders, and were treated more frequently with psychotropic drugs (Table 3). The prevalence of patients receiving OAT treated for more than 5 years with an OAT was higher in patients with chronic pain than in patients with no chronic pain (43.4 vs. 32.5%, *p* = 0.04). Finally, compared to patients receiving OAT with no chronic pain, patients receiving OAT with chronic pain had a higher average daily dose of buprenorphine [11.1 ± 7.1 (range: 0.8–32) vs. 8.9 ± 6.1 (range: 0.4–24) mg/day, *p* = 0.04], and split their OAT more frequently, specifically for pain management (24.3 vs. 6.9%, *p* = 0.009).

**Table 3:** Comparison of characteristics of patients receiving OAT with chronic pain vs. patients receiving OAT without chronic pain.

	**Chronic pain patients** ***n* = 169**	**Non chronic pain patients** ***n* = 340**	***P*-value**
	**%**	**%**	
**Age, mean ± SD [min-max]**	38.4 ± 9.1 [20–62]	36.1 ± 9.1 [19–64]	0.006
**Male gender**	76.3	78.5	0.57
**Unemployment**	65.7	52.0	0.003
**Type of OAT**			
Buprenorphine	36.1	30.9	0.24
Methadone	63.9	69.1	
**Duration of OAT**			
<1 year	16.8	16.2	0.04
1–5 years	39.8	51.3	
>5 years	43.4	32.5	
**Methadone dose (mg/day), mean ± SD [min-max]**	66.1 ± 38.3 [2–180]	62.5 ± 41.6 [3–300]	0.45
**Buprenorphine dose (mg/day), mean ± SD [min-max]**	11.1 ± 7.1 [0.8–32]	8.9 ± 6.1 [0.4–24]	0.04
**OAT splitting for pain**	24.3 (9)	6.9	0.009
**Psychotropic drug use**	50.3	39.1	0.02
**Alcohol use disorder treatment**	2.4	2.7	0.85
**Anxiolytic drugs**	34.3	23.2	0.008
**HIV**	3.7	3.0	0.57
**HCV**	23.8	16.9	0.09
**HBV**	4.9	5.1	0.29
**Tobacco**	88.8	87.4	0.64
**Alcohol consumption ≥ 3 glasses/day**	28.4	21.8	0.11
**SCL_6 score, mean ± SD [min-max]**	1.43 ± 0.98 [0–4]	0.87 ± 0.84 [0–3.5]	<0.0001
Anxiety	1.31 ± 1.07 [0–4]	0.74 ± 0.87 [0–4]	<0.0001
Depression	1.72 ± 1.21 [0–4]	1.13 ± 1.03 [0–4]	<0.0001
Psychoticism	1.25 ± 1.09 [0–4]	0.75 ± 0.97 [0–4]	<0.0001
**First consumption of illicit opioid in painful context**	11.8	7.4	0.10

*OAT, opioid agonist treatment; HIV, human immunodeficiency virus; HCV, hepatitis C virus; HBV, hepatitis B virus*.

### Analgesic Treatments and Drugs in Patients Receiving OAT With Chronic Pain

Among patients receiving OAT with chronic pain, 15.4% were not prescribed and did not self-medicate with any analgesic drugs, 52.1% were prescribed analgesics but almost half of them (48.9%) also self-medicated for pain management, and 32.5% exclusively self-medicated for pain management. Overall, 37.9% of patients receiving OAT with chronic pain achieved at least half of the maximum possible pain relief.

Among patients receiving OAT with chronic pain, non-opioid analgesics were prescribed more frequently than opioid analgesics, 47.3 vs. 21.9%. The most frequently used non-opioid analgesics were acetaminophen (68.8%), non-steroidal anti-inflammatory drugs (45.0%), and nefopam (4.7%). The most frequently used opioid analgesics were tramadol ± acetaminophen, 20.2%; codeine ± acetaminophen, 16.0%; and opium, 4.7%, and morphine, 11.2%; fentanyl, 1.2%; and oxycodone, 0.6%. Furthermore, 20.1% of patients receiving OAT with chronic pain also used illicit drugs for pain relief, including cannabis (15.0%), cocaine (3.0%), and street morphine (3.0%).

### Factors Associated With Chronic Pain in Patients Receiving OAT

In univariate analysis, general characteristics associated with chronic pain in patients receiving OAT were age [OR = 1.03 (95% CI: 1.01–1.05), *p* = 0.006], being unemployed [OR = 1.77 (1.20–2.60), *p* = 0.004], concomitant use of psychotropic drugs [OR = 1.58 (1.09–2.29), *p* = 0.02], SCL6-anxiety [OR = 1.80 (1.48–2.18), *p* < 0.0001], SCL6-depression [OR = 1.58 (1.34–1.87), *p* < 0.0001], and SCL6-psychoticism [OR = 1.59 (1.32–1.90), *p* < 0.0001]. Other covariates significant at the *p* < 0.25 level were buprenorphine maintenance treatment [reference = methadone, OR = 1.16 (0.86–1.87), *p* = 0.24], withdrawal-related pain during the last 7 days [OR = 1.57 (0.98–2.52), *p* = 0.06], alcohol consumption ≥ 3 glasses per day [OR = 1.42 (0.91–2.21), *p* = 0.12], and first consumption of illicit opioids in a painful context [OR = 1.68 (0.90–3.11), *p* = 0.10]. Other variables that have been tested but were not significantly associated with chronic pain in patients receiving OAT were gender, marital status, duration of OAT, HIV/HCV/HBV infection, tobacco use, OAT splitting, and concomitant use of illicit drugs.

**Figure 2:**
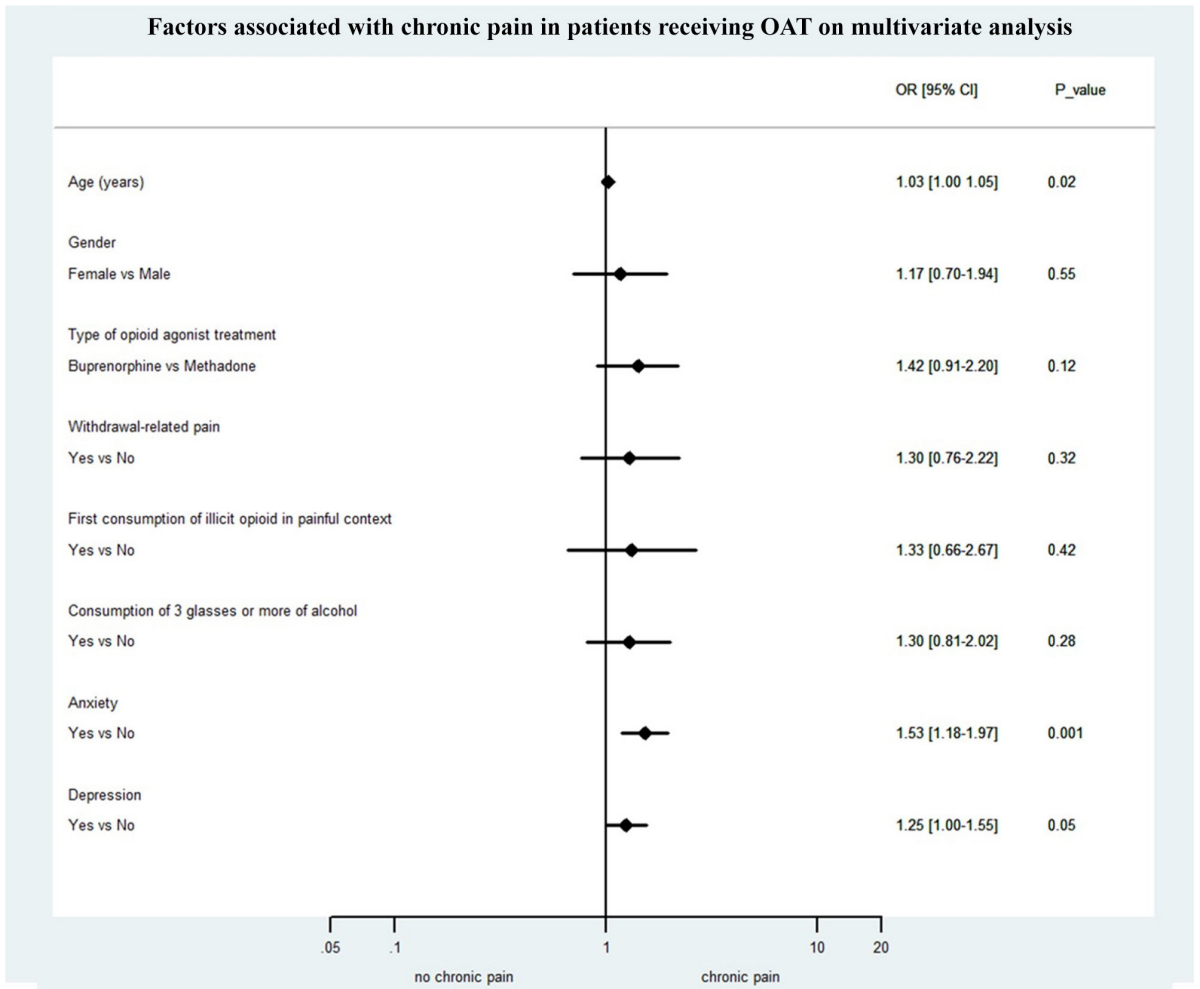
Factors associated with chronic pain on multivariate analysis.

On multivariate analysis (Figure 2), parameters that remained significantly associated with chronic pain were age [OR = 1.03 (1.00–1.05), *p* = 0.02], SCL6-anxiety [OR = 1.52 (95% CI: 1.15–2.02), *p* = 0.003], and SCL6-depression [OR = 1.25 (1.00–1.55), *p* = 0.05].

## Discussion

To the best of our knowledge, this French study is the first survey that provides insights into the prevalence of chronic pain in patients receiving OAT outside the US. The prevalence of chronic pain in patients receiving OAT was estimated at 33.2% (IC 95%: 29.1–37.3), with no difference between MMPs [31.5% (26.6–36.4)] and BMPs [36.8% (29.5–44.1)].

In the literature, few studies have focused on the prevalence of chronic pain in patients receiving OAT, with many differences in methodology or chronic pain presence and severity definitions. So any indirect comparison of estimates between studies remains challenging because of methodological issues and absence of an appropriate age- and sex-matched control population.

For chronic pain lasting at least 3 months, prevalence estimates in the literature vary from 41.9 to 68.0% in patients receiving OAT (6, 9), from 37.8 to 62.0% for methadone-treated patients (4, 6, 8), and from 36.0 to 43.3% for buprenorphine-treated patients (5, 6). According to Dunn et al., there was no statistically significant difference between prevalence of chronic pain in methadone and buprenorphine-maintained patients (6). Some other studies defined chronic pain as lasting for at least 6 months. In these cases, prevalence estimates ranged from 37 to 55.3% for methadone-treated patients (1–3) and was 48.5% for buprenorphine-treated patients (7).

Interestingly, the prevalence estimate in the present field study is very similar to that obtained in a recent data-based epidemiological study conducted by our team using the capture-recapture method on the comprehensive national French reimbursement database. In that work, the prevalence of chronic pain in patients receiving OAT ranged from 23.6% (14.9–46.2) to 32.1% (28.6–36.3). Moreover, compared to a sex- and age-matched control group of patients without OAT, the prevalence of chronic pain was 4-fold higher in patients receiving OAT (10). This finding is consistent with previous studies, which reported that chronic pain was more prevalent among patients receiving OAT than in the general population (1, 28).

Several hypotheses may account for these differences between patients receiving OAT and the general population. Compared to the general population, patients receiving OAT may display particular characteristics regarding pain, such as an opioid-induced hyperalgesia, where a state of increased sensitivity to painful stimuli is observed in patients receiving OAT (29–33), and a higher incidence of severe medical and psychiatric comorbidities that are well-known to be associated with chronic pain (34). Additionally, opioid withdrawal symptoms often include pain and may contribute to, or aggravate, chronic pain, therefore, it is important to identify this specific confounding painful condition and manage it with proper substitution and treatment dose adjustments. Inadequate treatment of pain is frequently observed in patients receiving OAT and this is in line with the results of the present study which has shown a number of important findings.

- Firstly, the prevalence of analgesics opioid prescription (excluding methadone/buprenorphine) was 2.2-fold lower than non-opioid prescription. Specifically, only 21.9% of patients receiving OAT with chronic pain were prescribed opioid analgesics, even though 75% of patients receiving OAT with chronic pain had moderate or severe chronic pain. Altogether, these findings may indicate either undertreatment or underdiagnosis.
- Secondly, only four out of 10 patients reported being effectively relieved of their chronic pain.
- Thirdly, among patients receiving OAT with prescribed analgesics, half of them self-medicated with additional analgesics, suggesting an undertreatment and insufficient relief. Several studies showed that 38.0–75.0% of patients receiving OAT with chronic pain also resorted to self-medication, including using over-the-counter drugs (1, 6, 13).
- Fourthly, almost one third of patients receiving OAT with chronic pain were not prescribed any analgesics and self-medicated. It can be speculated that the use of self-medication was intended to supplement the lack of analgesic prescribing. This may suggest the possible absence of a previous appropriate diagnosis of chronic pain by a physician or an underestimation of the intensity of chronic pain.
- Fifthly, one out of five patients used illicit drugs, such as cannabis, to relieve their chronic pain. In the literature, several studies have shown that the lack of pain treatment may encourage patients receiving OAT to use licit or illicit opioids, and other drugs such as benzodiazepines or cannabis, to self-treat their pain (1, 4, 6, 7, 12, 20, 28, 35, 36).

These pain-relief seeking behaviors may reflect an inadequate pain diagnosis and management. Some studies reported that practitioners were reluctant to prescribe opioid analgesics (opiophobia) because of concerns surrounding the risk of relapse, risk of misuse, and diversion, as well as the risk of overdose associated with opioid tolerance (37, 38). Of interest, the complex interaction between analgesic opioids, chronic pain, and opioid dependence has been illustrated in a study we conducted in 2018, which reported that patients receiving OAT with chronic pain received less analgesic opioid prescriptions than the general population (21). Currently, no guidelines specifically designed for pain management in patients receiving OAT are available. However, a recent and comprehensive review by Koller et al., proposed several practical suggestions to provide adequate and effective pain management in patients receiving OAT (39): in the first instance, authors propose to test elevation of opioid agonist treatment, dose splitting, or change of substitution; then they suggest regional analgesia, non-opioid analgesia, antidepressants and multimodal pain management (physiotherapy, heat treatment, acupuncture, biofeedback, and hypnosis); and finally, an additional opioid medication is possible as well.

As mentioned above, a number of comorbidities are associated with an increased prevalence of chronic pain in patients receiving OAT. In the present study, age, anxiety, and depression were clinical factors that were significantly associated with chronic pain in multivariate analysis. This is consistent with previous literature data where age (1, 28, 40) and psychiatric disorders are well-known to be associated with both chronic pain, and substance use disorders (1–3, 6, 12–14, 28, 41). In 2016, an American study reported a high rate of anxiety (52.0%) and mood disorders (57.0%) in patients with coexisting chronic pain and opioid use disorders (34). Dhingra et al. showed that half of methadone-maintained patients with chronic pain had moderate or severe depressive symptomatology (13).

The coexistence of both a mental health disorder and a substance use disorder is currently acknowledged to be a co-occurring disorder and refers to the concept of “dual diagnosis” (42, 43). The high prevalence of chronic pain adds a third potential clinical problem in patients receiving OAT. This “triple diagnosis” can be difficult to manage, owing to the complexity or severity of symptoms, and presents a considerable and real challenge for caregivers. Its effective management requires a comprehensive approach that recognizes the biological, pharmacological, social, and psychiatric aspects. Patient assessment should include a drug abuse history, evaluation of their mental state, and evaluation of their pain. In many cases, people receive treatment for one disorder, while the others remain under- or untreated. It is worth noting that when undiagnosed or untreated, one of these conditions could result in an imbalance in, or an aggravation of, the two other associated conditions. Finally, interdisciplinary management involving pain physicians, psychiatrists and addiction specialists needs to be implemented in order to manage patients with this “triple diagnosis” (44).

### Strengths and Limitations

This is the first multicentric study that has provided estimates of the prevalence of chronic pain in patients receiving OAT outside the US, which should bring some perspective to the field, both for France and across Europe. These findings should be useful to help clinicians gain awareness of the need to routinely identify and adequately manage chronic pain in patients receiving OAT. Moreover, the use of validated tools for measuring pain, psychological conditions, and general health make our results robust and reliable.

However, this study had several limitations. First, despite the limited sample size, our participants were representative of the larger population managed in specialized centers in France. In particular, the ratio of methadone to buprenorphine prescriptions was realistic, there was a high proportion of males, and the mean age was consistent (45–47). Yet, opioid substitution treatments are mainly prescribed in GP offices in France and GPs predominantly prescribe buprenorphine. Consequently, the patients receiving OAT of this study may be more representative of those in specialized centers, rather than of the whole population of patients receiving OAT in France. Secondly, these results were based on self-reported ratings which are prone to information bias, including recall bias. Thirdly, there was a lack of specific details regarding current pain treatment (indications, dosages) and etiology of chronic pain (musculoskeletal, neuropathic, and mixed). Finally, the alternative non-pharmacological strategies to relieve pain were not assessed in the present study.

## Conclusion

The prevalence of chronic pain in patients receiving OAT in this study was far from trivial. Chronic pain was often moderate to severe and interfered significantly with daily life activities. Of interest, the appropriate management of chronic pain in the present study remained uncertain, since insufficient relief and frequent additional self-medications with analgesics or illicit drugs were reported by patients receiving OAT. Given the number of identified barriers for a proper pain management, there is an urgent need to pay systematic attention to pain diagnosis and management in patients receiving OAT. In this context, the elaboration of specific training and guidelines that care-providers could refer to, as well as the development of structured and multidisciplinary pain management programs dedicated to patients receiving OAT, should be strongly encouraged. Finally, patient-focused management needs to be implemented, in the setting of an integrated care of both pain and addiction, to improve individual clinical outcomes along both domains.

## Data Availability Statement

The original contributions presented in the study are included in the article/supplementary material, further inquiries can be directed to the corresponding author/s.

## Ethics Statement

The studies involving human participants were reviewed and approved by Ethique committees CPP Sud-Est VI Clermont-Ferrand. Written informed consent for participation was not required for this study in accordance with the national legislation and the institutional requirements.

## Toxidol Study Group

Mohamed Allouach^1^, Jérome Bachelier^2^, Heiner Brinnel^3^, Philippe Dubost^4^, Hugues Leloup^5^, Van Phuc Nguyen^6^, Frédéric Plotka-Brun^7^, Mathilde Poirson^8^, Pierre Polomeni^9^, Christine Rouanet^10^, Sophie Velastegui^11^ and Pierre Villeger^12^

^1^ Équipe de Liaison et de Soins en Addictologie (ELSA), Centre Hospitalier de Givors, Givors, France

^2^ Centre de Soins et d'Accompagnement et de Prévention en Addictologie (CSAPA), centre Port-Bretagne, Tours, France

^3^ Centre de Soins et d'Accompagnement et de Prévention en Addictologie (CSAPA), Centre Hospitalier de l'Arbresle, BP116, L'Arbresle Cedex, France

^4^ Centre de Soins et d'Accompagnement et de Prévention en Addictologie (CSAPA), CH Moulins, Moulins, France

^5^ Centre de Soins et d'Accompagnement et de Prévention en Addictologie (CSAPA), Association Nationale de Prévention en Alcoologie et Addictologie (ANPAA), Issoire, France

^6^ Équipe de Liaison et de Soins en Addictologie (ELSA), Centre Hospitalier de Rouanne, Roanne, France

^7^ Centre de Soins et d'Accompagnement et de Prévention en Addictologie (CSAPA) “Fil à Fil”, Association Nationale de Prévention en Alcoologie et Addictologie (ANPAA), Montluçon, France

^8^ Centre de Soins et d'Accompagnement et de Prévention en Addictologie (CSAPA) “Danielle Casanova”, Marseille, France

^9^ Centre de Soins et d'Accompagnement et de Prévention en Addictologie (CSAPA), Hôpital René Muret, Sevran, France

^10^ Centre de Soins et d'Accompagnement et de Prévention en Addictologie (CSAPA), Association Nationale de Prévention en Alcoologie et Addictologie (ANPAA), Clermont-Ferrand, France

^11^ Centre d'Accueil et de Soins des conduites Addictives, CHU, Clermont de l'Oise, France

^12^ Centre de Soins et d'Accompagnement et de Prévention en Addictologie (CSAPA), CHU Limoges, Pôle Addictologie, Limoges, France

## Author Contributions

NA and CC developed the concept, devised the study, and reviewed the manuscript. JD takes responsibility for the integrity of the data, the accuracy of the data analysis, and wrote the first draft of the manuscript. J-LK and CC managed the literature searches and made substantial contributions to the data analysis. GB, MD, LP, J-PD, J-MD, PL, and AG provided revision of the intellectual content and final approval of the manuscript. All authors have participated sufficiently in the work to take responsibility for authorship and publication.

## Conflict of Interest

GB received sponsorship to attend scientific meetings, speaker honoraria, from Lundbeck, Merck-Lipha, Indivior, Bristol-Myers Squibb, Otsuka, Eutherapie, Sanofi Aventis, and AstraZeneca. MD received honoraria for speaking at conferences from Indivior, Recordati, and Camurus Laboratories, and honoraria for consultancy from Indivior, Camurus, and Accord Healthcare. LP received speaker honoraria from Recordati and Indivior laboratories. The remaining authors declare that the research was conducted in the absence of any commercial or financial relationships that could be construed as a potential conflict of interest.
